# Initial survey on the use of animals in scientific research and teaching reveals divided opinion of the Brazilian population

**DOI:** 10.31744/einstein_journal/2020AO5451

**Published:** 2020-11-05

**Authors:** Monica Levy Andersen, Lucile Maria Floeter-Winter, Sergio Tufik

**Affiliations:** 1 Universidade Federal de São Paulo São PauloSP Brazil Universidade Federal de São Paulo, São Paulo, SP, Brazil.; 2 Universidade de São Paulo São PauloSP Brazil Universidade de São Paulo, São Paulo, SP, Brazil.

**Keywords:** Animal ethics committees, Animal welfare, Animals, laboratory, Alternative methods, Legislation, 3R's principle

## Abstract

**Objective:**

Specific legislation regulating the use of animals in research in Brazil was introduced in 2008. However, the viewpoint of the Brazilian population regarding the use of animals in research and teaching activities remains largely unknown. Investigation of the public viewpoint on and understanding of the topic is required given the current shifts in the animal ethics scenario in Brazil. The objective of this study was to provide the first insight into the Brazilian population viewpoint on the use of animals in scientific research and teaching activities.

**Methods:**

Data collected in a survey involving 2,115 individuals aged 16 years or older and residing in 130 municipalities distributed across the five Brazilian macroregions (North, Northeast, South, Southeast, and Midwest) were analyzed. The margin of error for entire sample was set at 2%, with a 95% confidence interval.

**Results:**

This survey revealed that most Brazilian citizens are in favor of the use animals in research, particularly for medical purposes. Different views depending on the nature of research were identified. Approximately 80% of respondents were also in favor of frequent oversight of laboratories and animal facilities.

**Conclusion:**

Survey findings indicate that the opinion of the Brazilian population is divided when it comes to the use of animals in scientific research and teaching. Divided opinions expose a limited understanding of the importance of basic sciences and emphasizes the need for improved communication between the scientific community and the general population. Further strategies aimed to promote animal welfare are discussed.

## INTRODUCTION

In medicine and biology, researchers often resort to animal models as an alternative to human experimentation. This practice stemmed from more than a century of scientific development, during which alternatives to human testing were sought out. Animal models, such as rats, mice, zebrafish, *Drosophila melanogaster* and many others emerged during this process, albeit not without controversy. As science became an increasingly larger part of common knowledge, public opinion on human and animal testing began to take shape, often with a firm stance against experimentation on living organisms.^(^^1^^)^

In response to shifts in public opinion, scientific societies, governments and regulatory agencies worldwide began to give serious thought to the ethical aspects of animal testing in science. Laws, directives and regulatory bodies have been created to govern the use of animals in the central, regional and local/institutional spheres in several countries.^(^^1^^)^ These measures often follow guidelines aimed at minimizing animal suffering, such as replacement of animal testing whenever possible (henceforth referred to as “alternative methods to animal use” – AMA), reduction of the number of animals used where their use is unavoidable and refinement of experimental methods to mitigate or avoid animal pain and distress.^(^^1^^,^^2^^)^

In Brazil, the introduction of legislation regulating the use of animals in scientific research was a long process. After years of debate at the National Congress, the issue was finally addressed in law 11.794/2008, published on October 8, 2008.^(^^3^^)^ This law, known as Arouca Law, established criteria for “*criação e uso de animais para fins de ensino e pesquisa em todo o território nacional*”. Hence, since 2008 there is legal framework for standardization and regulation of animal use in scientific research and teaching, including the application of legal and administrative sanctions should its provisions be violated. This advancement emphasized the significance of animal welfare, thenceforth regulated by law and binding criteria in Brazil.

In 2009, the Arouca Law also provided the creation of the *Conselho Nacional de Controle de Experimentação Animal* (CONCEA). This body includes representatives of the government, scientific community, pharmaceutical industry and animal protection societies, and has regulatory, advisory, decision-making and appeal roles.

The law published in 2008 also determined the creation of Ethics Committees on Animal Use (CEUA - *Comissão de Ética no Uso de Animais*) in organizations that breed or use vertebrate animals for teaching or scientific purposes.^(^^3^^)^ Decree no. 6899/2009, issued for complementary regulation and amendment of the Arouca Law, gave rise to the *Cadastro das Instituições de Uso Científico de Animais* (CIUCA). This decree dictates that organizations using animals in their activities must record all data pertaining to their respective animal facilities in the CIUCA, and be registered and licensed by CONCEA. Deposition of data in the CIUCA facilitates licensing and inspection procedures and contributes to the delineation of a national profile.^(^^4^^)^

Law 11.794/2008 has existed for over a decade. Still, little is known about the viewpoint of the Brazilian population on animal use in research or teaching activities. Awareness of the Brazilian public opinion regarding ethical aspects of animal experimentation is essential for public policy debate improvement and development and may assist CONCEA in decision making in consonance with scientific community requirements as well as public opinion.

National surveys may provide insights into public opinion on the use of laboratory animals in health applications, such as vaccine and new drug development. Animal welfare has received increasing attention in surveys worldwide and in studies investigating the topic in different groups, including medicine and veterinary medicine students, researchers and professors in bioscience and biomedical areas and the general population.^(^^5^^-^^8^^)^ Also, professional training accounting for ethical aspects of animal use will ensure well-trained professionals perform their activities within ethical boundaries.^(^^9^^)^ Therefore, investigation of the Brazilian public opinion on animal use and experimentation may contribute useful information to lawmakers and regulatory agencies for future policy-making.

## OBJECTIVE

To provide the first insight into the Brazilian population viewpoint on the use of animals in scientific research and teaching activities.

## METHODS

### Survey procedures

A representative sample of the Brazilian population was surveyed by *DataFolha* research institute. To ensure representativeness, sampling was carried out according to the 2010 national census conducted by the Brazilian Institute of Geography and Statistics (IBGE - *Instituto Brasileiro de Geografia e Estatistica*).^(^^10^^)^ Sample design accounted for distribution according to gender and age, size of municipality (metropolitan or urban areas) and geographic region (North/Midwest, Northeast, Southeast and South). Sample design included the following stages: stratification of the general population according to federal unit and size of municipality; selection of target municipalities; randomized selection of survey sites per municipality; and selection of interviewees according to age and gender quotas.

Interviews were conducted in person using a defined questionnaire administered via tablet computers. Participants were invited to express their personal opinion on animal use in research and teaching.

Participants were asked six questions addressing the aforementioned issues. Predefined answers were “in favor” or “against”. Answers such as “I do not care”, “Maybe” and “I do not know” were not encouraged (*i.e*., were spontaneously provided) but were recorded if given and included in the data analysis. Survey questions are listed in table 1.

**Table 1:** Questions carried out about the animal use in research and teaching

“Regarding the use of animals in different situations, are you in favor or against _____ (read each entry below)?”
1) Frequent inspection of laboratories and facilities where animals are bred or used for research and testing?
2) The use of animals in research and testing for vaccine development against diseases, such as Zika, dengue and others?
3) The use of animals in research and tests that may contribute, even if indirectly, to find a cure for diseases?
4) The use of alternative methods to animal use in research, testing and teaching?
5) The use of animals in basic science research and testing?
6) The use of animals in wet lab sessions in schools and universities?

Questions encompassed the use of animals in different types of research (vaccine development, disease cure development and basic sciences) and in teaching activities. The use of AMA in such activities and whether facilities using animals should be frequently inspected were also interrogated.

### Sample

A total of 2,115 interviews were conducted in 130 municipalities across Brazil. Sample size was calculated by *DataFolha* research institute to give a margin of error of 2% with a 95% confidence level, while representing the Brazilian population aged ≥16 years. To ensure overall representativeness, the sample was weighted by gender, age, size of municipality and geographical distribution.

### Data collection period

Data collection was carried out between November 30 and December 6, 2016.

### Data quality controls

Data quality was controlled by *DataFolha* research institute as follows: after in-person interviews using a tablet computer, data were checked for consistency via telephone calls. Each interviewer called at least 20% of their interviewees. Internal consistency of questionnaires and database was also checked.

### Statistical analysis

Data analysis was based on ratios and measures of central tendency such as mean and median values. Data interpretation was limited to differences greater than the margin of error between results.

### Ethical statement

Public opinion surveys with non-identified participants are exempted from ethical approval by the National Ethics Committee on Research (CONEP – *Comissão Nacional de Ética em Pesquisa).*

## RESULTS

This sample comprised primarily women (52%). Age was normally distributed (mean age, 42 years). Most interviewees had completed primary or secondary education (39% and 45% respectively) and two-thirds had children (2.5 children/couple on average). Most (63%) respondents were economically active at the time of interview, with higher prevalence of salaried employees (25%).

**Figure 1:**
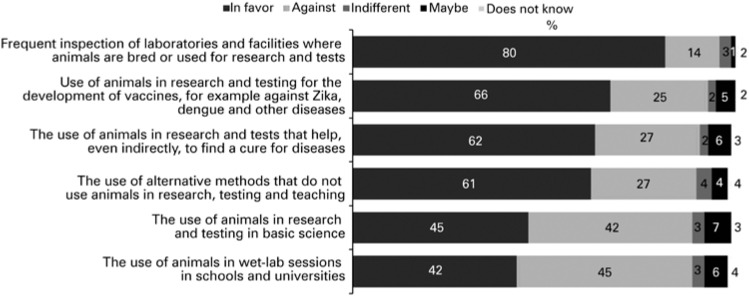
Viewpoints on the use of animals in scientific research, experimental testing and teaching activities (n=2,115 respondents)

Most respondents had a personal stance on animal use in research, with a small proportion (2% to 4%) reporting indifference, lack of a particular opinion or indecision. Oversight of facilities where animals are bred and kept for testing was the item enjoying strongest support among the Brazilian population (80%; Figure 1). Use of animals for vaccine development or in tests that may contribute, even if indirectly, to find a cure for diseases also enjoyed significant support (66% and 62%, respectively). Search for AMA in research, testing and teaching was supported by 61% of respondents.

Conversely, use of animals in basic science research and testing aroused mixed opinions (45% of respondents in favor and 42% against), as did use of animals in wet lab sessions in schools and universities (42% in favor and 45% against). Survey findings extrapolated to the entire Brazilian population aged 16 or over (158.161.107 individuals) are shown in table 2.^(^^11^^)^

**Table 2:** Projected estimated population in favor of using animals in different research and testing settings

Survey question	Respondents in favor (%)	Projected estimated population (numbers, in millions)
Frequent inspection of laboratories and facilities where animals are bred or used for research and testing	80	127
Use of animals in research and testing for vaccine development against diseases such as Zika, dengue and others	66	104
Use of animals in research and tests that may contribute, even if indirectly, to find a cure for diseases	62	98
Alternative methods to animal use in research, testing and teaching	61	96
Use of animals in basic science research and testing	45	71
Use of animals in wet lab sessions in schools and universities	42	66

Table 3 shows respondents’ viewpoints according to socioeconomic status. Use of animals for vaccine development or in research that may contribute, even if indirectly, to find a cure for diseases was widely accepted among men (72% and 70%, respectively), while women were less in favor of the use of animals for vaccine development (60%), and even less in research that may contribute, even if indirectly, to find a cure for diseases (55%). Approval of laboratory oversight increased in direct proportion to level of education and economic status. Individuals opposed to the use of animals for vaccine development or in research that may contribute, even if indirectly, to find a cure for diseases prevailed in the 16-24-year-old group. This and the 25-34-year-old group were the most supportive of AMA in research (64% and 69%, respectively). Use of AMA also enjoyed greater support among respondents with higher socioeconomic status and level of education.

**Table 3:** Respondents’ answers to survey questions according to gender, age, schooling and economic class

		Total	Gender		Age (years)					Schooling			Economic bracket		
			Male	Female	16-24	25-34	35-44	45-69	≥60	Primary	Secondary	Tertiary	Class A/B	Class C	Class D/E
Frequent inspection of laboratories															
	In favor	80	82	78	77	82	80	81	78	76	81	87	85	79	76
	Against	14	12	15	19	13	15	12	11	15	14	9	11	15	15
	Indifferent	3	2	3	2	3	3	2	3	3	2	2	2	3	2
	Maybe	1	1	2	2	1	1	1	3	2	1	2	1	1	2
Use of animals for vaccine development															
	In favor	66	72	60	60	68	63	68	67	66	64	67	65	64	69
	Against	25	20	30	32	25	27	23	20	23	27	25	26	27	21
	Indifferent	2	2	2	2	2	3	1	3	2	2	1	2	3	1
	Maybe	5	4	6	6	4	6	4	5	4	5	7	7	5	4
Use of animals in research that may contribute, even if indirectly, to find a cure for diseases															
	In favor	62	70	55	61	63	63	61	62	61	63	63	63	60	64
	Against	27	21	32	31	28	27	25	23	25	28	26	27	28	24
	Indifferent	2	2	3	1	3	2	2	3	3	2	3	2	4	1
	Maybe	6	5	7	6	4	5	7	7	6	5	8	7	5	5
Alternative methods to animal use in research															
	In favor	61	61	61	64	69	60	57	57	58	62	69	68	60	57
	Against	27	28	26	28	24	29	29	24	27	29	20	21	27	30
	Indifferent	4	4	4	3	3	5	4	7	5	4	4	4	5	3
	Maybe	4	3	4	4	3	4	5	3	3	3	6	5	4	3
Use of animals in basic science research and testing															
	In favor	45	53	38	40	49	43	46	47	48	44	42	43	44	48
	Against	42	35	48	50	43	43	39	33	39	44	42	44	42	39
	Indifferent	3	3	4	1	3	2	3	6	4	3	2	3	4	3
	Maybe	7	7	7	7	4	10	8	8	5	7	13	10	7	5
Use of animals in wet lab sessions in schools and universities															
	In favor	42	50	35	38	47	40	40	46	43	42	43	43	42	42
	Against	45	39	50	52	43	47	46	36	42	47	45	46	46	43
	Indifferent	3	3	3	1	3	3	2	5	3	3	1	2	3	4
	Maybe	6	5	7	6	6	7	7	5	5	6	10	8	6	5
Respondents (n)		2,115	1,036	1,079	455	553	372	436	299	751	1,004	360	524	1,019	572

Results expressed as % if not reported otherwise.

Margin of error: 2%.

Table 4 shows respondents’ viewpoints according to geographic region. Individuals living in the South were the least supportive of frequent inspection of laboratories (75% compared to ≥80% in remaining regions). However, the proportion of individuals that were against inspection was similar across regions (13% to 16%). This difference reflects indifferent or undecided respondents in the South. Support to the use of animals for vaccine development and in research that may contribute, even if indirectly, to find a cure for diseases was greater among individuals living in the Northeast Region or in urban areas relative to their respective counterparts (other regions or metropolitan areas). Use of animals in basic science was more strongly opposed by respondents living in the Southeast (45% compared to 40% in favor), whereas individuals living in the remaining regions were more in favor of than opposed to this practice. Use of animals in wet lab sessions also enjoyed greater support in the South, Northeast and North/Midwest (≥44%) relative to the Southeast (39%) Region. Opposition to this practice was lowest among individuals living in the South (38%) compared to other regions and lower among individuals residing in urban compared to metropolitan areas (43% and 47%, respectively).

**Table 4:** Respondents’ answers to survey questions according to geographic region

		Total	Geographic region				Type of municipality	
			Southeast	South	Northeast	North/Midwest	Metropolitan	Urban
Frequent inspection of laboratories								
	In favor	80	81	75	80	81	81	79
	Against	14	13	14	16	14	13	14
	Indifferent	3	2	6	2	3	3	3
	Maybe	1	2	2	1	1	1	1
Use of animals for vaccine development								
	In favor	66	61	60	74	69	61	69
	Against	25	27	28	21	23	29	23
	Indifferent	2	2	3	0	4	2	2
	Maybe	5	7	5	3	2	6	4
Use of animals in research that may contribute, even if indirectly, to find a cure for diseases								
	In favor	62	60	55	70	63	59	64
	Against	27	28	28	23	29	30	24
	Indifferent	2	2	5	−	3	3	2
	Maybe	6	7	7	4	3	6	5
Alternative methods to animal use in research								
	In favor	61	63	63	58	61	62	61
	Against	27	24	21	33	30	27	27
	Indifferent	4	3	8	3	4	5	4
	Maybe	4	6	5	2	1	4	4
Use of animals in basic science research and testing								
	In favor	45	40	46	49	51	43	46
	Against	42	45	38	40	40	43	40
	Indifferent	3	3	6	2	5	3	3
	Maybe	7	10	7	5	3	8	7
Use of animals in wet lab sessions in schools and universities								
	In favor	42	39	45	45	44	41	43
	Against	45	45	38	46	48	47	43
	Indifferent	3	3	6	1	3	3	3
	Maybe	6	8	7	5	4	6	6
Respondents (n)		2,115	885	308	535	387	923	1,192

Results expressed as % if not reported otherwise.

Margin of error = 2%.

Zero (0): less than 0.5%; dash (-): no answer.

## DISCUSSION

This survey was the first to reveal the Brazilian population viewpoint on the use of animals in different types of scientific research and teaching activities. Respondents answers to six questions interrogating the use of animals in different settings revealed mixed opinions. The use of animals in applied sciences (*e.g*., research aimed at finding a cure for diseases or developing vaccines) enjoyed significant support, whereas the use of animals in basic science research and wet lab sessions elicited more objection and greater percentages of answers such as indifferent or undecided.

Mixed opinions were also reported in industrialized countries such as Germany, Belgium, Italy, England, Ireland, Denmark, and Spain.^(^^12^^)^ Animal use in research enjoyed over 50% support in Portugal (65%) and Greece (64%), whereas most people in France (68%) stood against this practice. Outside Europe, Japanese and Canadian citizens also expressed mixed opinions, with more than 50% supporting animal use.^(^^12^^)^ According to a study conducted by Pew Research Center and the American Association for the Advancement of Science, North-American citizens are also divided over the use of animals in scientific research (47% in favor and 50% against). In striking contrast, the same survey revealed that 89% of members of the scientific community support the use of animals in research.^(^^13^^)^ These findings unveil a gap between the public at large and the scientific community when it comes to animal testing, at least in the United States.

In this study, eight out of ten respondents were in favor of monitoring of animal use in laboratories and facilities using or breeding these animals for scientific purposes, suggesting public support for oversight of animal use by regulatory agencies.

Half of female and 39% of male respondents were against the use of animals in wet labs. There is no obvious explanation for this finding, but similar results have been reported elsewhere.^(^^12^^,^^14^^)^ In this regard, Brazilian institutions have recently adapted to legal provisions determining the implementation of CEUAs. However, adaptation of existing infrastructure may be limited by lack of funding.

This survey revealed that more than half of the population (61%) is in favor of AMA in research. In 2016, CONCEA organized the Symposium on Alternative Methods to the Use of Animals in Teaching, in which methods currently used to replace animals in scientific and teaching activities across the country were presented.

Also in 2016, CONCEA published the *Diretriz Brasileira para o Cuidado e a Utilização de Animais em Atividades de Ensino ou de Pesquisa Científica* (DBCA). This document addressed institutional responsibilities regarding the provision of AMA in teaching and established the conscientious objection policy (Act 5.1.1 of DBCA, Normative Resolution n. 30), which now has ethical and legal binding. This policy dictates that students have the right to opt for AMA in research and should be encouraged to seek their validation. The application of AMA in teaching is more complex than in research settings, given few organizations are currently capable of implementing them. Validated guidelines for evaluating teaching are available.^(^^15^^-^^18^^)^ However, new approaches are often difficult to communicate or implement.

In Brazil, several organizations are involved in the validation of AMA in experimental procedures, namely the *Centro Brasileiro para Validação de Métodos Alternativos* (BraCVAM) and the *Rede Nacional de Métodos Alternativos ao Uso de Animais* – (Renama). Together, these organizations work to raise awareness about the applicability of AMA and their benefits to researchers, students and teachers. Once validated, the alternative method is regulated by CONCEA, which then establishes a deadline for total replacement of animal use.

Among several regulatory guidelines published by CONCEA, some address the recognition of AMA in scientific research. It should be noted that CONCEA works in alignment with the National Agency of Health Surveillance (Anvisa - *Agência Nacional de Vigilância Sanitária*) that has also published a resolution addressing the adoption of AMA (RDC 35/2015) in response to CONCEA guidelines, in which 24 AMA used in experimental testing of substances with known results are detailed.

### Future strategies

Based on viewpoints highlighted in this survey, the following recommendations for the scientific community, funding, regulatory and advisory agencies and oversight organizations can be made:

1. Development of work plans for improved animal facility oversight;
2. Foster information exchange between CONCEA and CEUAs;
3. Promotion of AMA training courses for students, researchers and technicians;
4. Provision of financial support to research investigating animal use and AMA;
5. Actions aimed to promote increased understanding of basic and applied sciences among the general population;
6. And actions aimed to bridge the gap between the viewpoint of the scientific community and the general public on animal use.

The last two items could be achieved via educational and awareness campaigns.

### Limitations

Statistical analysis of public opinion surveys is notoriously complex and there no such thing as a single correct manner to perform it. Also, given the size of the population represented, even minimal differences in percentages in the survey can translate into millions of people in the real world - not a negligible amount. For these reasons, data analysis in this study was limited to differences greater than the margin of error in results. While simplistic, this approach is statistically valid and yields meaningful data.

Another limitation is related to question 5, “Regarding the use of animals in different situations, are you in favor or against the use of animals in basic science research and testing?”. The term “basic science” may be misinterpreted by the lay public. However, in public opinion questionnaires, questions must be phrased so as not to induce particular answers or response bias. Therefore, this question was worded to keep it as neutral as possible.

## CONCLUSION

The viewpoint of the Brazilian population on the use of animals in scientific research and teaching is divided. Only a small portion of respondents declared being indifferent, not having a particular opinion about the practice or being undecided. Less than half were in favor of the use of animals in basic science research and wet labs and over 60% supported the use of alternative methods to animal use. In contrast, respondents largely supported the use of animals in research associated with practical benefits such as vaccine development and cure for diseases.

## References

[1] 1. Gauthier C, Griffin G. Using animals in research, testing and teaching. Rev Sci Tech. 2005;24(2):735-45. Review. 16358523

[2] 2. Ormandy EH, Schuppli CA, Weary DM. Worldwide trends in the use of animals in research: the contribution of genetically-modified animal models. Altern Lab Anim. 2009;37(1):63-8. 10.1177/026119290903700109 19292576

[3] 3. Brasil. Presidência da República. Lei n. 11.794, de 8 de outubro de 2008. Regulamenta o inciso VII do § 1o do art. 225 da Constituição Federal, estabelecendo procedimentos para o uso científico de animais; revoga a Lei no 6.638, de 8 de maio de 1979; e dá outras providências [Internet]. Brasília (DF): Presidência da República do Brasil; 2008 Out 8 [citado 2020 Jun 9]. Disponível em: http://www.planalto.gov.br/ccivil_03/_ato2007-2010/2008/lei/l11794.htm

[4] 4. Brasil. Presidência da República. Decreto 6.899, de 15 de julho de 2009. Dispõe sobre a composição do Conselho Nacional de Controle de Experimentação Animal - CONCEA, estabelece as normas para o seu funcionamento e de sua Secretaria-Executiva, cria o Cadastro das Instituições de Uso Científico de Animais - CIUCA, mediante a regulamentação da Lei no 11.794, de 8 de outubro de 2008, que dispõe sobre procedimentos para o uso científico de animais, e dá outras providências [Internet]. Brasília (DF): Presidência da República do Brasil; 2009 Jul 15 [citado Jun 9]. Disponível em: https://www2.camara.leg.br/legin/fed/decret/2009/decreto-6899-15-julho-2009-589524-norma-pe.html

[5] 5. Dohn NB, Fago A, Overgaard J, Madsen PT, Malte H. Students’ motivation toward laboratory work in physiology teaching. Adv Physiol Educ. 2016; 40(3):313-18. 10.1152/advan.00029.2016 27445278

[6] 6. Crettaz von Roten F. Laboratory animal science course in Switzerland: participants’ points of view and implications for organizers. Lab Anim. 2018; 52(1):69-78. 10.1177/0023677217708807 28571490

[7] 7. Dilly M, Read EK, Baillie S. A Survey of Established Veterinary Clinical Skills Laboratories from Europe and North America: Present Practices and Recent Developments. J Vet Med Educ. 2017;44(4):580-9. 10.3138/jvme.0216-030R1 28534722

[8] 8. Magnani D, Ferri N, Dalmau A, Messori S. Knowledge and opinions of veterinary students in Italy toward animal welfare science and law. Vet Rec. 2017;180(9):225. 10.1136/vr.103938 28174218

[9] 9. Iki Y, Ito T, Kudo K, Noda M, Kanehira M, Sueta T, et al. Animal ethics and welfare education in wet-lab training can foster residents’ ethical values toward life. Exp Anim. 2017;66(4):313-20. 10.1538/expanim.17-0026 PMC5682343 28592716

[10] 10. Instituto Brasileiro de Geografia e Estatistica (IBGE). Censo Demográfico 2010. Características da população e dos domicílios. Resultados do universo [Internet]. Rio de Janeiro: IBGE; 2011 [citado 2020 Jun 9]. Disponível em: https://biblioteca.ibge.gov.br/visualizacao/periodicos/93/cd_2010_caracteristicas_populacao_domicilios.pdf

[11] 11. Instituto Brasileiro de Geografia e Estatistica (IBGE). Pesquisa Nacional por Amostra de Domicílios: síntese de indicadores 2015 [Internet]. Rio de Janeiro (RJ): IBGE; 2016 [citado 2020 Jun 9]. Disponível em: https://biblioteca.ibge.gov.br/visualizacao/livros/liv98887.pdf

[12] 12. Pifer L, Shimizu K, Pifer R. Public attitudes toward animal research: some international comparisons. Soc Anim. 1994;2(2):95-113. 10.1163/156853094x00126 11654358

[13] 13. Funk C, Rainie L, Page D. Public and Scientists’ Views on Science and Society [Internet]. Washington (DC): Pew Research Center; 2015 [cited 2020 June 9]. Available from: https://www.pewresearch.org/science/2015/01/29/public-and-scientists-views-on-science-and-society/

[14] 14. Wells DL Hepper PG. Pet ownership and adults’ views on the use of animals. Soc Anim. 1997;5(1):45-63.

[15] 15. Van Amburgh JA, Devlin JW, Kirwin JL, Qualters DM. A tool for measuring active learning in the classroom. Am J Pharm Educ. 2007;71(5):85. 10.5688/aj710585 PMC2064883 17998982

[16] 16. Creating Effective Teaching and Learning Environments (OECD). First Results from Teaching And Learning International Survey (TALIS) [Internet]. Paris (FR); 2009 [cited 2020 Oct 20]. Available from: http://www.oecd.org/education/school/43023606.pdf

[17] 17. Fluit CR, Bolhuis S, Grol R, Laan R, Wensing M. Assessing the quality of clinical teachers: a systematic review of content and quality of questionnaires for assessing clinical teachers. J Gen Intern Med. 2010;25(12):1337-45. Review. 10.1007/s11606-010-1458-y PMC2988147 20703952

[18] 18. Pat-El RJ, Tillema H, Segers M, Vedder P. Validation of Assessment for Learning Questionnaires for teachers and students. Br J Educ Psychol. 2013;83(1):98-113. 10.1111/j.2044-8279.2011.02057.x 23369177

