# Supplementary material for: Initial survey on the use of animals in scientific research and teaching reveals divided opinion of the Brazilian population
Source: Einstein (Sao Paulo). 2020 Nov 5;18:eAO5451. doi: 10.31744/einstein_journal/2020AO5451 (PMC7647387; doi:10.31744/einstein_journal/2020AO5451)
Supplement: Translation

# Translation: Investigação inicial sobre o uso de animais na pesquisa científica e no ensino revela opinião dividida da população brasileira

**Authors:** Monica Levy Andersen, Lucile Maria Floeter-Winter, Sergio Tufik

**Affiliations:** 1 São PauloSP Brasil Universidade Federal de São Paulo, São Paulo, SP, Brasil.; 2 São PauloSP Brasil Universidade de São Paulo, São Paulo, SP, Brasil.

**Keywords:** Comitês de ética animal, Bem-estar do animal, Animais de laboratório, Métodos alternativos, Legislação, Princípio dos 3Rs

## Abstract

**Objetivo:**

A legislação específica que regula o uso de animais em pesquisa no Brasil foi introduzida em 2008. No entanto, a opinião da população brasileira sobre o uso de animais em atividades de pesquisa e ensino ainda é desconhecida. No atual cenário brasileiro em mudança com relação à ética animal, é necessário avaliar as visões e o conhecimento da população sobre o assunto. O objetivo deste destudo foi realizar o primeiro levantamento da opinião da população brasileira sobre o uso de animais em atividades de ensino e pesquisa científica.

**Métodos:**

Analisamos os resultados de uma pesquisa com 2.115 indivíduos com 16 anos ou mais de 130 municípios das cinco macrorregiões brasileiras (Norte, Nordeste, Sul, Sudeste e Centro-Oeste). A margem de erro para toda a amostra foi de 2% dentro de um intervalo de confiança de 95%.

**Resultados:**

A pesquisa revelou que a maioria da população brasileira era favorável ao uso de animais em pesquisas, principalmente para fins médicos. Diferentes pontos de vista, dependendo da natureza da pesquisa, também foram identificados. Além disso, aproximadamente 80% dos entrevistados eram favoráveis ao monitoramento frequente de laboratórios e instalações de animais.

**Conclusão:**

A opinião da população brasileira está dividida com relação ao uso de animais em pesquisa e ensino científicos. Essa divisão expõe um entendimento limitado da importância das ciências básicas e destaca a necessidade de uma melhor comunicação entre a comunidade científica e a população em geral. Outras ações para alcançar as melhorias desejadas no bem-estar animal são discutidas.

## INTRODUÇÃO

Nas áreas médica e biológica, os pesquisadores, muitas vezes, lançam mão de modelos animais como alternativa à experimentação direta em seres humanos. Essa prática resultou de mais de um século de desenvolvimento científico, período marcado pela busca de alternativas à experimentação em seres humanos. Modelos animais, como ratos, camundongos, peixe-zebra (paulistinha ou *zebrafish*), *Drosophila melanogaster* e muitos outros surgiram ao longo desse processo, porém não sem controvérsia. Conforme a ciência se tornou uma parte cada vez maior do conhecimento comum, a opinião pública a respeito da experimentação em seres humanos e animais adquiriu forma, muitas vezes com postura fortemente contrária à experimentação em seres vivos.^(^^1^^)^

Em resposta à opinião pública, as sociedades científicas, o governos e as agências reguladoras do mundo todo passaram a considerar seriamente os aspectos éticos envolvidos na experimentação científica em animais. Diversos países criaram leis, instruções normativas e órgãos regulatórios voltados ao uso de animais nas esferas central, regional e local/institucional.^(^^1^^)^ Essas medidas geralmente seguem diretrizes que determinam a minimização do sofrimento animal por meio de sua substituição sempre que possível (daqui em diante referida como “métodos alternativos ao uso de animais” – AMA), a redução do número de animais quando seu uso for inevitável e o refinamento da metodologia experimental, a fim de mitigar ou evitar a dor e o sofrimento dos animais.^(^^1^^,^^2^^)^

No Brasil, a introdução da legislação referente ao uso de animais na pesquisa científica foi um longo processo. Depois de anos de discussão no Congresso Nacional, a questão foi finalmente abordada na lei 11.794/2008, publicada em 8 de outubro de 2008.^(^^3^^)^ Conhecido como Lei Arouca, o texto estabelece critérios para “criação e uso de animais para fins de ensino e pesquisa em todo o território nacional”. Assim, desde 2008 existe um arcabouço legal para a padronização e a regulamentação do uso de animais no ensino e na pesquisa científica, assim como sanções legais e administrativas para ações que violem suas disposições. Esse avanço reforçou a importância do bem-estar animal, que, desde então, passou a ser regulamentado por lei e critérios de observância obrigatória no Brasil.

Em 2009, a Lei Arouca também determinou a criação do Conselho Nacional de Controle de Experimentação Animal (CONCEA). Esse conselho é formado por representantes do governo, da comunidade científica, da indústria farmacêutica e das sociedades protetoras e tem papel regulatório, recomendatório, decisório e de apelação.

A lei publicada em 2008 também determinou a criação da Comissão de Ética no Uso de Animais (CEUA) em instituições que criam ou utilizam animais vertebrados para fins educativos ou científicos.^(^^3^^)^ O decreto 6899/2009, que regulamentou e emedou a Lei Arouca, também levou à criação do Cadastro das Instituições de Uso Científico de Animais (CIUCA). Esse decreto exige que as instituições que utilizam animais em suas atividades documentem toda a informação referente às respectivas instalações de manutenção e uso no CIUCA, além de determinar a obrigatoriedade de obtenção de registro e anuência junto ao CONCEA. A disponibilização de dados no CIUCA facilita os procedimentos de concessão de licença e inspeção e contribui para o delineamento de um perfil nacional.^(^^4^^)^

Embora a lei 11.794/2008 já exista há mais de uma década, pouco se sabe sobre a opinião da população brasileira a respeito do uso de animais em atividades de ensino ou pesquisa. O conhecimento da opinião pública brasileira sobre os aspectos éticos envolvidos na experimentação animal é fundamental para a discussão, a melhoria e o desenvolvimento de políticas públicas, e pode ajudar o CONCEA na tomada de decisões que reflitam tanto as necessidades da comunidade científica como a opinião pública.

Pesquisas nacionais podem revelar a opinião da população sobre o uso de animais de laboratório em procedimentos de saúde, como o desenvolvimento de vacinas e novas drogas. A preocupação com o bem-estar animal foi tema de pesquisas no mundo todo e de estudos que avaliaram aspectos relacionados em diferentes grupos, incluindo os estudantes de medicina e medicina veterinária, os pesquisadores e professores da área de biociências e biomédica e a população em geral.^(^^5^^-^^8^^)^ Além disso, o treinamento profissional, que leva em conta os aspectos éticos do uso de animais, garante que profissionais bem treinados realizem suas funções dentro de limites éticos.^(^^9^^)^ Desse modo, o levantamento da opinião pública brasileira sobre o assunto pode fornecer informações úteis para legisladores e agências reguladoras, no que se refere ao direcionamento de políticas futuras.

## OBJETIVO

Realizar o primeiro levantamento da opinião da população brasileira sobre o uso de animais em atividades de ensino e pesquisa científica.

## MÉTODOS

### Elaboração da pesquisa

Uma amostra representativa da população brasileira foi entrevistada pelo instituto de pesquisa DataFolha. A fim de garantir a representatividade, a amostragem foi realizada de acordo com o censo nacional de 2010 do Instituto Brasileiro de Geografia e Estatística (IBGE).^(^^10^^)^ O delineamento amostral considerou a distribuição da população por gênero e idade, tamanho do município (áreas metropolitanas ou urbanas) e região geográfica (Norte/Centro-Oeste, Nordeste, Sudeste e Sul). O delineamento amostral foi realizado de acordo com as seguintes etapas: estratificação da população em geral por unidade federativa e tamanho do município; seleção de municípios-alvo; seleção aleatória de locais de aplicação da pesquisa em cada município e seleção de entrevistados por cota de idade e sexo.

As entrevistas foram realizadas de forma presencial, por meio de um questionário definido, administrado por tablet. Cada participante foi convidado a expressar sua opinião sobre o uso de animais no ensino e na pesquisa.

Os participantes foram interrogados por meio de seis perguntas relacionadas ao tema, sendo as respostas pré-definidas “a favor” ou “contra”. As respostas “não me importo”, “talvez” e “não sei” não foram estimuladas (os participantes responderam de forma espontânea), mas forma registradas quando fornecidas e incluídas na análise de dados. As perguntas feitas encontram-se listadas na tabela 1.

**Tabela 1:** Perguntas realizadas em pesquisa sobre o uso de animais em atividades de ensino e pesquisa científica

“No que se refere ao uso de animais em diferentes situações, você é a favor ou contra _____ (leia cada uma das entradas abaixo)?”
1) A inspeção frequente de laboratórios e instalações de criação e uso de animais destinados à pesquisa e à experimentação?
2) O uso de animais em pesquisas e experimentação destinadas ao desenvolvimento de vacinas, contra Zika, dengue e outras doenças, for exemplo?
3) O uso de animais em pesquisas e experimentação que contribuam, ainda que indiretamente, para a busca de cura para doenças?
4) O uso de métodos alternativos que dispensam o uso animais em pesquisa, experimentação ou ensino?
5) O uso de animais em pesquisa e experimentação em ciências básicas?
6) O uso de animais em aulas práticas em escolas e universidades?

As perguntas englobaram o uso de animais em diferentes tipos de pesquisa (desenvolvimento de vacinas, desenvolvimento de cura para doenças e ciências básicas) e atividades de ensino, além do uso de AMA e a questão da inspeção frequente das instalações e dos locais de uso.

### Amostra

Foram realizadas 2.115 entrevistas em 130 municípios brasileiros. O tamanho amostral foi calculado pelo instituto de pesquisa DataFolha com margem de erro de 2% e intervalo de confiança de 95% para representação da população brasileira com idade ≥16 anos. A fim de garantir a representatividade, a amostra foi ponderada por sexo, idade, tamanho do município e distribuição geográfica.

### Período de coleta de dados

Os dados foram coletados entre 30 de novembro e 6 de dezembro de 2016.

### Controle de qualidade de dados

O controle de qualidade de dados foi realizado pelo instituto de pesquisa DataFolha, de acordo com a seguinte metodologia: após a pesquisa presencial por tablet, a coerência dos dados coletados foi verificada por telefone. Cada entrevistador ligou para pelo menos 20% de seus entrevistados. Todos os questionários e bancos de dados foram verificados quanto à coerência interna.

### Análise estatística

A análise dos dados foi baseada em medidas estatísticas de proporções e tendência central, como médias e medianas. Os dados foram interpretados considerando-se diferenças entre resultados superiores à margem de erro.

### Declaração de ética

Pesquisas de opinião pública sem identificação de participantes são isentas da necessidade de aprovação pela Comissão Nacional de Ética em Pesquisa (CONEP).

## RESULTADOS

A amostra de entrevistados foi composta majoritariamente por mulheres (52%). A idade apresentou distribuição normal, com média de 42 anos. A maioria dos entrevistados tinha Ensino Fundamental (39%) ou Médio (45%) completo. Dois terços dos entrevistados tinham filhos (média de 2,5 filhos/casal), e a maioria (63%) era economicamente ativa na época da entrevista, com maior prevalência de assalariados (25%).

A maioria dos respondentes tinha uma opinião formada a respeito do uso de animais em pesquisa e poucos (2% a 4%) se declararam indiferentes ou indecisos. O monitoramento das instalações de criação e manutenção de animais para testes foi o item com maior apoio por parte da população brasileira (80%; Figura 1). O uso de animais no desenvolvimento de vacinas ou testes que pudessem contribuir, ainda que indiretamente, para a cura de doenças também teve aprovação significativa (66% e 62%, respectivamente). A busca por AMA em atividades de pesquisa, experimentação e ensino teve o apoio de 61% dos respondentes.

**Figura 1:** Opiniões sobre o uso de animais em pesquisa científica, testes experimentais e atividades de ensino (n=2.115 respondentes)

Ao contrário, o uso de animais em pesquisa e experimentação em ciências básicas despertou opiniões divergentes, com 45% dos respondentes declarando-se a favor e 42% contra. Opiniões divergentes também foram manifestadas quanto às aulas práticas em escolas e universidades, com 42% dos respondentes declarando-se a favor e 45% contra. A tabela 2 mostra os resultados da pesquisa extrapolados para toda a população brasileira, com idade igual ou superior a 16 anos (158.161.107 indivíduos).^(^^11^^)^

**Tabela 2:** População estimada projetada a favor do uso de animais em diferentes tipos de pesquisa e experimentação

Pergunta da pesquisa	Respondentes a favor (%)	População estimada projetada (números em milhões)
Inspeção frequente de laboratórios e instalações de criação e uso de animais destinados à pesquisa e à experimentação	80	127
O uso de animais em pesquisa e experimentação destinadas ao desenvolvimento de vacinas, contra Zika, dengue e outras doenças, for exemplo	66	104
O uso de animais em pesquisas e experimentação que contribuam, ainda que indiretamente, para a busca de cura para doenças	62	98
Métodos alternativos ao uso de animais em pesquisa, teste e ensino	61	96
O uso de animais em pesquisa e experimentação em ciências básicas	45	71
O uso de animais em aulas práticas em escolas e universidades	42	66

A tabela 3 mostra a opinião da amostra de acordo com a condição socioeconômica. O uso de animais para o desenvolvimento de vacinas ou em pesquisas que possam contribuir, ainda que indiretamente, para a cura de doenças teve ampla aceitação entre os homens (72% e 70%, respectivamente). Já as mulheres se mostraram menos favoráveis ao uso de animais para o desenvolvimento de vacinas (60%) e menos ainda em pesquisas que pudessem contribuir, ainda que indiretamente, para a cura de doenças (55%). O apoio à supervisão de laboratórios aumentou de forma proporcional ao nível educacional e econômico dos respondentes. Indivíduos contrários ao uso de animais para o desenvolvimento de vacinas ou em pesquisas que pudessem contribuir, ainda que indiretamente, para a cura de doenças, predominaram na faixa etária de 16 a 24 anos. Indivíduos dessa faixa etária, somada aos de 25 a 34 anos idade foram os mais favoráveis ao uso de AMA em pesquisa (64% e 69%, respectivamente). O uso de AMA também gozou de amplo suporte entre respondentes de classes socioeconômicas e nível educacional mais elevados.

**Tabela 3:** Respostas dadas pelos respondentes às perguntas da pesquisa, distribuídas por sexo, idade, nível educacional e classe econômica

		Total (%)	Sexo		Idade (anos)					Nível educacional			Nível socioeconômico		
			Masculino	Feminino	16-24	25-34	35-44	45-69	≥60	Primária	Secundária	Terciária	Classe A/B	Classe C	Classe D/E
Inspeção frequente de laboratórios															
	A favor	80	82	78	77	82	80	81	78	76	81	87	85	79	76
	Contra	14	12	15	19	13	15	12	11	15	14	9	11	15	15
	Indiferente	3	2	3	2	3	3	2	3	3	2	2	2	3	2
	Talvez	1	1	2	2	1	1	1	3	2	1	2	1	1	2
Uso de animais para desenvolvimento de vacinas															
	A favor	66	72	60	60	68	63	68	67	66	64	67	65	64	69
	Contra	25	20	30	32	25	27	23	20	23	27	25	26	27	21
	Indiferente	2	2	2	2	2	3	1	3	2	2	1	2	3	1
	Talvez	5	4	6	6	4	6	4	5	4	5	7	7	5	4
Uso de animais em pesquisas que possam contribuir, ainda que indiretamente, para a cura de doenças															
	A favor	62	70	55	61	63	63	61	62	61	63	63	63	60	64
	Contra	27	21	32	31	28	27	25	23	25	28	26	27	28	24
	Indiferente	2	2	3	1	3	2	2	3	3	2	3	2	4	1
	Talvez	6	5	7	6	4	5	7	7	6	5	8	7	5	5
Métodos alternativos que dispensam o uso de animais em pesquisa															
	A favor	61	61	61	64	69	60	57	57	58	62	69	68	60	57
	Contra	27	28	26	28	24	29	29	24	27	29	20	21	27	30
	Indiferente	4	4	4	3	3	5	4	7	5	4	4	4	5	3
	Talvez	4	3	4	4	3	4	5	3	3	3	6	5	4	3
Uso de animais e pesquisa e experimentação em ciências básicas															
	A favor	45	53	38	40	49	43	46	47	48	44	42	43	44	48
	Contra	42	35	48	50	43	43	39	33	39	44	42	44	42	39
	Indiferente	3	3	4	1	3	2	3	6	4	3	2	3	4	3
	Talvez	7	7	7	7	4	10	8	8	5	7	13	10	7	5
Uso de animais em aulas práticas em escolas e universidades															
	A favor	42	50	35	38	47	40	40	46	43	42	43	43	42	42
	Contra	45	39	50	52	43	47	46	36	42	47	45	46	46	43
	Indiferente	3	3	3	1	3	3	2	5	3	3	1	2	3	4
	Talvez	6	5	7	6	6	7	7	5	5	6	10	8	6	5
Respondentes (n)		2.115	1.036	1.079	455	553	372	436	299	751	1.004	360	524	1.019	572

Resultados expressos como % quando não informado de outra forma.

Margem de erro: 2%.

A tabela 4 mostra a opinião da amostra por região demográfica. Indivíduos do Sul mostraram-se menos favoráveis à inspeção frequente de laboratórios (75% contra ≥80% nas demais regiões). Entretanto, a proporção de indivíduos contrários à inspeção foi semelhante em todas as regiões (13% a 16%). A diferença reflete os respondentes indiferentes ou indecisos do Sul. Os residentes da Região Nordeste ou de áreas urbanas mostraram-se mais favoráveis ao uso de animais para desenvolvimento de vacinas e busca de cura para doenças do que seus respectivos pares (residentes de outras regiões ou áreas metropolitanas). O uso de animais em ciências básicas encontrou oposição mais forte entre os respondentes da Região Sudeste (45% contra e 40% a favor), enquanto indivíduos de outras regiões mostraram-se mais favoráveis de que contrários a essa prática. O uso de animais em aulas práticas também gozou de maior apoio nas Regiões Sul, Nordeste e Norte/Centro-Oeste (≥44%) do que na Região Sudeste (39%). A oposição a essa prática foi menor no Sul do que nas demais regiões (38%). Residentes de áreas metropolitanas também se mostraram mais contrários a essa prática do que os de áreas urbanas (47% contra 43%).

**Tabela 4:** Respostas dadas pelos respondentes às perguntas da pesquisa, distribuídas por região geográfica

		Total	Região geográfica				Tipo de município	
			Sudeste	Sul	Nordeste	Norte/Centro-Oeste	Metropolitano	Urbano
Inspeção frequente de laboratórios								
	A favor	80	81	75	80	81	81	79
	Contra	14	13	14	16	14	13	14
	Indiferente	3	2	6	2	3	3	3
	Talvez	1	2	2	1	1	1	1
Uso de animais para desenvolvimento de vacinas								
	A favor	66	61	60	74	69	61	69
	Contra	25	27	28	21	23	29	23
	Indiferente	2	2	3	0	4	2	2
	Talvez	5	7	5	3	2	6	4
Uso de animais em pesquisas que possam contribuir, ainda que indiretamente, para a cura de doenças								
	A favor	62	60	55	70	63	59	64
	Contra	27	28	28	23	29	30	24
	Indiferente	2	2	5	−	3	3	2
	Talvez	6	7	7	4	3	6	5
Métodos alternativos que dispensam o uso de animais em pesquisa								
	A favor	61	63	63	58	61	62	61
	Contra	27	24	21	33	30	27	27
	Indiferente	4	3	8	3	4	5	4
	Talvez	4	6	5	2	1	4	4
Uso de animais e pesquisa e experimentação em ciências básicas								
	A favor	45	40	46	49	51	43	46
	Contra	42	45	38	40	40	43	40
	Indiferente	3	3	6	2	5	3	3
	Talvez	7	10	7	5	3	8	7
Uso de animais em aulas práticas em escolas e universidades								
	A favor	42	39	45	45	44	41	43
	Contra	45	45	38	46	48	47	43
	Indiferente	3	3	6	1	3	3	3
	Talvez	6	8	7	5	4	6	6
Respondentes (n)		2.115	885	308	535	387	923	1.192

Resultados expressos como % quando não informado de outra forma.

Margem de erro = 2%.

Zero (0): menos de 0,5%; traço (-): sem resposta.

## DISCUSSÃO

Esta pesquisa foi a primeira a revelar a opinião da população brasileira sobre o uso de animais em diferentes tipos de atividades de pesquisa científica e ensino. As respostas fornecidas às seis perguntas sobre o uso de animais nessas circunstâncias revelaram opiniões mistas, com respostas favoráveis ao uso de animais em ciências aplicadas, como pesquisas destinadas à busca pela cura de doenças e à produção de vacinas, e maior oposição ao uso de animais em ciências básicas e no ensino – áreas estas que também tiveram maior percentagem de respostas como “indiferente” ou “indeciso”.

Pesquisas em países industrializados, como Alemanha, Bélgica, Itália, Inglaterra, Irlanda, Dinamarca e Espanha, também revelaram opiniões mistas.^(^^12^^)^ O uso de animais gozou de mais de 50% de apoio em Portugal (65%) e na Grécia (64%), enquanto a maior parte da população (68%) se mostrou contrária a essa prática na França. Fora da Europa, opiniões mistas também foram manifestadas por cidadãos do Japão e do Canadá, com mais de 50% de apoio ao uso de animais.^(^^12^^)^ Um estudo realizado pelo *Pew Research Center* e pela *American Association for the Advancement of Science* revelou que os cidadãos norte-americanos também se mostram divididos quanto ao uso de animais na pesquisa científica: 47% a favor e 50% contra. Em contraste gritante, a mesma pesquisa mostrou que 89% da comunidade científica é a favor do uso de animais em pesquisa.^(^^13^^)^ Esses achados expõem a lacuna que separa a opinião pública da opinião da comunidade científica no que se refere à experimentação animal, pelo menos nos Estados Unidos.

Neste estudo, oito de cada dez respondentes mostraram-se a favor do monitoramento do uso de animais em laboratórios e instituições que usam ou criam animais para fins científicos, sugerindo apoio público à supervisão do uso de animais por agências reguladoras.

Metade das mulheres e 39% dos homens que responderam à pesquisa mostraram-se contrários ao uso de animais em aulas práticas. Embora não haja uma explicação óbvia para esse achado, resultados semelhantes foram relatados em outros estudos.^(^^12^^,^^14^^)^ No que se refere a essa questão, recentemente, as instituições brasileiras tiveram que se adaptar à determinação legal de implementação das CEUAs. Entretanto, a adaptação da infraestrutura existente pode ser limitada por falta de verba.

Esta pesquisa revelou que mais da metade da população (61%) é a favor do uso de AMA em pesquisa. Em 2016, o CONCEA organizou o Simpósio sobre Métodos Alternativos ao Uso de Animais no Ensino, no qual foram apresentados os métodos vigentes no país para substituir o uso de animais em atividades científicas e de ensino.

Ainda em 2016, o CONCEA publicou a Diretriz Brasileira para o Cuidado e a Utilização de Animais em Atividades de Ensino ou de Pesquisa Científica (DBCA). Esse documento aborda as responsabilidades institucionais relativas à implementação de AMA no ensino e estabelece a política de objeção consciente (Ato 5.1.1 da DBCA, Resolução Normativa No. 30), agora com obrigatoriedade ética e legal. Essa política confere aos estudantes o direito de optar por AMA em pesquisas e estimula a busca de sua validação. A aplicação de AMA no ensino é mais complexa do que em pesquisa, uma vez que poucas organizações se encontram devidamente preparadas para implementá-lo de imediato. Apesar da existência de algumas diretrizes para avaliação do ensino validadas,^(^^15^^-^^18^^)^ novas abordagens nem sempre são fáceis de comunicar e implementar.

No Brasil, diversas organizações estão empenhadas na validação de AMA em procedimentos experimentais, principalmente o Centro Brasileiro para Validação de Métodos Alternativos (BraCVAM) e a Rede Nacional de Métodos Alternativos ao Uso de Animais (Renama). Juntas, essas organizações promovem a conscientização a respeito da aplicabilidade de AMA e de seus benefícios para pesquisadores, estudantes e professores. Uma vez validado, o método passa a ser regulamentado pelo CONCEA, que estabelece um prazo para a substituição total do uso de animais.

Dentre as diversas diretrizes regulatórias publicadas pelo CONCEA, algumas abordam o reconhecimento de AMA na pesquisa científica. Cabe notar que o CONCEA trabalha em conjunto com a Agência Nacional de Vigilância Sanitária (Anvisa), que também publicou resolução sobre a adoção de AMA (Resolução da Diretoria Colegiada 35/2015) em resposta às diretrizes do CONCEA, detalhando 24 AMA utilizados em testes experimentais de substâncias com resultados conhecidos.

### Estratégias futuras

Em vista das opiniões destacadas nesta pesquisa, recomendamos que a comunidade científica, as agências de fomento, reguladoras e recomendatórias e as organizações supervisórias desenvolvam planos de trabalho que melhorem a supervisão de instituições que criam e usam animais:

1. Estimulem a troca de informações entre o CONCEA e as CEUAs.
2. Promovam treinamentos em AMA destinados a estudantes, pesquisadores e técnicos;
3. Forneçam apoio financeiro a pesquisas sobre o uso de animais e AMA;
4. Fomentem ações que promovam o entendimento do significado dos termos ciência básica e ciência aplicada por parte da população em geral;
5. Disponibilizem ações no sentido de preencher a lacuna entre a opinião da comunidade científica;
6. E opinião pública a respeito do uso de animais.

Os dois últimos itens poderiam ser atendidos por meio de campanhas educacionais e de conscientização.

### Limitações

A análise estatística de sondagens de opinião pública é notoriamente complexa e não há uma única forma correta de realizá-la. Além disso, dado o tamanho da população representada, diferenças mínimas de percentagem na pesquisa podem se traduzir em milhões de pessoas na vida real, quantidade essa nada negligenciável. Por esses motivos, a análise dos dados apresentados neste estudo foi limitada às diferenças superiores à margem de erro nos resultados. Embora simplista, essa abordagem é estatisticamente válida e fornece informações valiosas.

Outra limitação diz respeito à pergunta 5, “No que se refere ao uso de animais em diferentes situações, você é a favor ou contra o uso de animais em pesquisa e experimentação em ciências básicas?”. Aqui, o termo “ciências básicas” pode ser interpretado de forma equivocada pelo público leigo. Entretanto, ao se elaborar um questionário destinado à sondagem da opinião pública, as perguntas devem ser formuladas de forma a não induzir respostas ou influenciar o respondente. A terminologia usada na pergunta em questão foi selecionada para torná-la o mais neutra possível.

## CONCLUSÃO

A opinião da população brasileira a respeito do uso de animais em pesquisa científica e no ensino é dividida. Apenas uma pequena proporção de respondentes se declarou indiferente, não soube responder ou se mostrou indecisa. Menos da metade foi a favor do uso de animais em ciências básicas e aulas práticas e a maioria mostrou-se a favor do uso de métodos alternativos. Por outro lado, o uso de animais em pesquisas associadas a benefícios práticos, como vacinas ou cura de doenças, gozou de amplo apoio entre os respondentes.
